# The Physician and His Journals

**Published:** 1889-10

**Authors:** 


THE PHYSICIAN AND HIS JOURNALS.—The *Journal of the American Medical Association*, in its issue of September 21, 1889, under the above title, discusses a subject that is of much importance, and does it in an able and interesting manner.

In these busy days, when time must be economized to the utmost degree, it is important that a physician's literature shall be carefully selected, with a view to yield the greatest benefit from a minimized outlay of cerebration and money,—of brain-cells and dollars.

In this selection, the *Journal* justly enjoins upon physicians, as a first duty, the support of and contribution to their home periodical, in the following well-chosen sentences :

There is an ever-present tendency among men, in a sort of vague and misty way, to venerate the past; in a way quite egotistical to magnify the present, and also to compass the future with grandiloquent interrogations.

To the first and third counts we plead not guilty,—but to the second we enter our confession, since a simple statement of facts can hardly seem to those in other lands other than most notable exhibitions of egotism. When we come to enumerate the medical practitioners in the United States, the number of our medical schools and their improved facilities for teaching, the rapid development of medical societies and the numbers of medical journals that are in demand, we submit that the parallel is not to be found in the past or present history of any nation. If any one has doubts upon this subject, we commend to him the simple study of statistics. Again, there never was a time when in this and in other lands there was such an array of talent applied with utmost tension, to medical investigation. There was never a time when there was so much of original discovery, and never before such facilities for rapid advances in medical education. There never was a time when medical journalism was so enterprising as now, nor its pages so filled with valuable instruction, and there never was a time when a physician could so soon fall behind and be lost sight of as now. A single year's neglect will render his needs conspicuous. Only the most industrious and critical readers are fully abreast of the times, and in the hour of need and of their opportunity, how quickly these come to commanding prominence.

. . . . . . . . . .

We believe that a first duty of the medical man is to help develop, foster, and sustain the medical societies and medical interest of his own locality. He has power personally to stimulate his associates, and to aid them in organization and in medical progress. In the development of such local interests, nothing can be so helpful as the ably-conducted and well-supported local medical journal. To this he owes a primary obligation, both literary and pecuniary. Its pages should be replete with the recorded experiences of local contributors, while, in turn, it should garner for them the best of medical productions from all lands.

To the physician in quest of his second journal, we respectfully recommend the value and the claims of the *Journal of the American Medical Association.* While it is national in its relations and circulation, it will seek to further his local interests. It will give a continued series of valuable original papers, prepared and submitted by the leading men of the profession, at the annual meetings of the American Medical Association. It will give prominence to translations of the best literature published in foreign languages, and copious selections from the best writings at home and abroad. It will devote itself to the dissemination of clinical instruction, and to the presentation of condensed reports of the proceedings of prominent medical societies. These, with the work of a corps of editorial writers widely representative of the American States, constitute our claim to the second place on the physician's list of medical journals, to which he should add as many others, home and foreign, as he can *thoroughly utilize.*

